# CHO-K1 cell-derived scFv targeting pseudorabies virus ameliorates multi-organ viremia and improves animal survival in a lethal infection model

**DOI:** 10.3389/fmicb.2026.1893255

**Published:** 2026-08-10

**Authors:** Yanmei Yang, Jiayu Yue, Jingying Xie, Xiangrong Li, Yan Cui, Ruofei Feng

**Affiliations:** 1 Department of Basic Veterinary Medicine, Faculty of Veterinary Medicine, Gansu Agricultural University, Lanzhou, China; 2 Department of Clinical Veterinary Medicine, Faculty of Veterinary Medicine, Gansu Province Livestock Embryo Engineering Research Center, Gansu Agricultural University, Lanzhou, China; 3 Key Laboratory of Biotechnology and Bioengineering of State Ethnic Biomedical Research Center, Northwest Minzu University, Lanzhou, China; 4 School of Life Sciences and Engineering, Northwest Minzu University, Lanzhou, China

**Keywords:** antibody therapy, antiviral, CHO-K1 cells, pseudorabies virus, scFv

## Abstract

The pseudorabies virus, with enhanced virulence, increased transmissibility, and severe co-infections, have re-emerged worldwide, leading to significant economic losses in swine industry. However, the traditional Bartha-K61 vaccine could not provide full protection against the PRV variant strains, thus, an urgent need for the development of novel prophylactic and therapeutic strategies. Here, we proposed a novel antibody construct, PRV single chain fragment variable (PRV-scFv), for PRV antibody therapy. The PRV-scFv genes were constructed by joining the *PRV-VH* and *PRV-VL* sequences, isolated from the immunized mice’s spleen, via a flexible (Gly4Ser)3 linker. This construct was stably expressed in CHO-K1 cells cultured in serum-free suspension. Based on these findings, we screened a monoclonal cell line, CHO-K1-PRV-scFv-2^E8^ cell, to robustly express PRV-scFv antibody that effectively inhibited PRV infection *in vitro*, reduced the viral load *in vivo*, and improved the survival rate of infected mice, which provided new insights into antibody therapy and veterinary therapeutic strategies development.

## 1 Introduction

Pseudorabies (PR) is an acute infectious disease caused by the pseudorabies virus (PRV) (Klupp et al., 2004; Lin et al., 2020; Ye et al., 2022). PRV is classified within the herpesvirus family, the alpha herpesvirus subfamily, and the genus varicella virus, and it is characterized as a linear double-stranded DNA virus with a genome approximately 143 kb in length (Ding et al., 2025). In the 1970s, the introduction of the Bartha-K61 gene-deficient attenuated vaccine achieved effective control of PRV spread in China (Luo et al., 2014). Although live-attenuated or inactivated vaccines are effective against classical PRV, they are challenged by the field virus strains. Developing new vaccines against these variants requires lengthy development cycles. Their production depends on infectious viruses, which raises biosecurity concerns, and the process of attenuation may increase the risks of reversion or inadequate immunity (Freuling et al., 2017; Nie et al., 2023; Sun et al., 2016; Wang et al., 2014; Wozniakowski and Samorek-Salamonowicz, 2015; Yin et al., 2025). Current live-attenuated or inactivated vaccines, while effective against classical PRV strains, face challenges from emerging field variants that may exhibit antigenic drift or immune evasion.

In addition, evidence suggests that some of these PRV variants may be linked to rare infections in humans, thereby raising public health concerns (Tong et al., 2015; Zhao et al., 2024; Zheng et al., 2019; Zhou et al., 2019). A more comprehensive understanding of the immune inhibition mechanisms employed by PRV is essential for the development of recombinant antibody and more effective strategies for disease control.

Single-chain fragment variables (scFv) represent a novel class of genetically engineered antibodies (Alfthan et al., 1995). These molecules are created by linking the variable heavy chain domain (VH) with the variable light chain domain (VL) using a peptide linker. ScFv antibodies exhibit characteristics such as enhanced affinity, solubility, multi-domain structure, and specific binding to target antigens. They have versatile applications in antibody engineering, biotechnology, oncology, as well as in antiviral research and diverse biomedical sectors. With their small size, stability, and low immunogenicity, scFv antibodies present innovative approaches for therapeutic antibodies, diagnostic tools, virus detection, treatment and prevention strategies.

In this study, we aimed to construct and characterize a PRV-specific scFv antibody expressed in CHO-K1 cells and evaluate its antiviral efficacy *in vitro* and *in vivo*. Specifically, we constructed a eukaryotic expression vector of PRV-scFv, utilizing inactivated Bartha-K61 attenuated strains to immunize BALB/c mice. The *PRV-VH* and *PRV-VL* genes were cloned from spleen RNA using a (Gly4Ser)3 linker. The PRV-scFv was expressed in CHO-K1 suspension culture cells and scFv with higher secretion expression were screened. To explore the efficiency of scFv for the inhibition of PRV infection *in vitro* and reduction of viral proliferation in multiple organs, a lethal PRV mice infection model was tested. Our data reveals that PRV-scFv antibody has great potential for PRV prevention and control, offering valuable insights for the development of new treatment methods and antibodies.

## 2 Materials and methods

### 2.1 Cells, viruses, bacterial strains, and animals

CHO-K1-S40 suspension culture cells (maintained by the Biomedical Research Center of Northwest Minzu University) were cultured in SFM4CHO (HyClone, Logan, USA) at 120 rpm in a 37 °C incubator with 5% CO_2_. BHK-21 cells (ATCC CCL-10) were cultured in DMEM (Bailing, Lanzhou, China) at 37 °C in a 5% CO_2_ incubator, supplemented with 10% NBS (new bovine serum). The PRV Bartha-K61 is an attenuated vaccine strain and stored in our laboratory. The competent cells of *Escherichia coli* BL21 were preserved and provided by the Biomedical Research Center of Northwest Minzu University. Male BALB/c mice were purchased from the Lanzhou Veterinary Research Institute, Chinese Academy of Agricultural Sciences. Animal welfare and experimental procedures were performed in strict accordance with the Guidelines for the Management and Use of Laboratory Animals (Ministry of Science and Technology of China, 2006) and approved by the Animal Ethics Committee of Northwest Minzu University and the Animal Protection and Utilization Committee (NO. XBMU-SM-20248).

### 2.2 Antibodies and chemical reagents

HRP-conjugated Goat Anti-Rabbit IgG (D110058), HRP-conjugated Rabbit anti-mouse IgG (D110098) and HRP-conjugated Goat Anti-Mouse IgG (D110087) were bought from Sangon Biotech (Shanghai, China). Pseudorabies Virus Polyclonal Antibody (PA1-081) were obtained from Thermo Fisher Scientific (Waltham, USA). *β*-actin Polyclonal antibody (20536-1-AP) and His-tag Polyclonal antibody (10001-0-AP) were acquired from Proteintech (Wuhan, China).

Pro Taq Hs Premix Probe qPCR Kit II (AG11712) purchased from Accurate Biology (Hunan, China). Amaxa™ SF Cell Line 4D-Nucleofector™ x Kit L (V4XC-2024) were obtained from Lonza (Basel, Switzerland). ELISA Blocking Kit for Detecting Pseudorabies Virus gB Antibody (20230201) were acquired from Wuhan Keqian Biological (Wuhan, China).

### 2.3 Determination of mice immunity and antibody levels

Six to eight-week-old male BALB/c mice were chosen for immunization with the attenuated strain of porcine pseudorabies Bartha-K61, which has been inactivated by a 30 min exposure to a 56 °C water bath, via subcutaneous and intramuscular injection. The initial immunization consisted of mixing the antigen with complete Freund’s adjuvant (CFA) in a 1:1 ratio, followed by the administration of 200 mL of the resulting emulsion. This was succeeded by three additional immunizations using incomplete Freund’s adjuvant at 14-day intervals. Mice were euthanized by gradual displacement with CO_2_ at a rate of 50% of the chamber volume per minute until respiratory arrest. Post-immunization, blood samples were collected, and serum was obtained by centrifuging at 3000 rpm for 10 min for ELISA analysis of PRV antibody levels following the manufacturer’s instructions according to the ELISA Blocking Kit for Detecting Pseudorabies Virus gB Antibody, and the S/N value was calculated according to the following formula. Spleens from PRV antibody-positive mice were harvested and stored at −80 °C for future use.

$$S/N=\frac{\text{Sample}\;\;OD_{630\mathrm{nm}}\;\;\text{value}}{\text{Negative}\;\;\text{control}\;\;OD_{630\mathrm{nm}}\;\;\text{average}\;\;\text{value}}\;\;$$

### 2.4 Cloning and identification of the variable domain gene of single-chain fragment variables

Primers *PRV-VH* F/R and *PRV-VL* F/R were designed to amplify the variable region genes of mouse antibody light and heavy chains (Table 1). Total RNA was extracted from the spleens of PRV antibody-positive mice using the TRIzol method. Following the protocol of the *Evo*-MLV RT-PCR Reverse Transcription Kit, total RNA was reverse-transcribed into cDNA. Subsequently, PCR amplification of the *PRV-VH* and *PRV-VL* genes was conducted using cDNA as the template and the *PRV-VH* F/R and *PRV-VL* F/R primers, respectively. The *PRV-VH* and *PRV-VL* genes were then cloned into the pMD18-T Vector. Single colonies were selected for PCR identification. The PCR-positive bacterial strains were sent to Tsingke Biotechnology Co., Ltd. (Xi’an, China) for sequencing.

**Table 1:** Primers used in this study (Italic indicates restriction enzyme sites).

Primer name	Primer sequence (5′-3′)
*PRV-VH* R	CG*GGATCC*ATGGAGGTSMARCTGCAGSAGTCWGG
*PRV-VH* F	CG*GAATTC*TGAGGAGACGGTGACCGTGGTCCCTTGG
*PRV-VL* R	CG*GGATCC*ATGGACATTGAGCTCACCCAGTCTCCA
*PRV-VL* F	CG*GAATTC*CCGTTTTATTTCCAACTTTGTССС

### 2.5 Phylogenetic analysis of variable region gene sequences

The variable region gene sequences of the obtained antibodies were analyzed for the *PRV-VH* and *PRV-VL* gene sequences using CLUSTALX. Subsequently, phylogenetic trees for the *PRV-VH* and *PRV-VL* genes were constructed with MEGA 5.10 software, employing the Neighbor-joining phylogenetic method.

### 2.6 Expression vector construction of PRV-VH and PRV-VL, cell transfection and western blotting

Phylogenetic analysis revealed three distinct *PRV-VH* and *PRV-VL* genes, each exhibiting relatively distant phylogenetic relationships, which were subsequently ligated to the pcDNA3.1(+)-C-6His vector. The recombinant plasmids were confirmed through double digestion with *EcoR* I and *BamH* I in a water bath maintained at 37 °C. A total of 2 × 10^6^ CHO-K1-S40 cells in six-well plates were transfected with the specified expression plasmids using the Lonza 4D-Nucleofector System. The CHO-K1 cells were cultured in SFM4CHO medium and continuously agitated at 120 rpm in a 37 °C incubator with 5% CO_2_ for 48 h. Cells were collected and lysed with RIPA lysis buffer (Solarbio, Beijing, China) containing phenylmethanesulfonyl fluoride (PMSF) for 20 min on ice. The lysates were then centrifuged at 12,000 rpm for 20 min. Culture supernatants were aliquoted, and equal amounts of cell extracts were denatured, subjected to 10% SDS-PAGE, and transferred to polyvinylidene fluoride (PVDF) membranes. The membranes were blocked with 2.5% skimmed milk in Tris-buffered saline and Tween 20 (TBST) for 2 h at room temperature. Subsequently, they were incubated with His-Tag Monoclonal antibody and Beta Actin Monoclonal antibody (1,5,000 dilution) for 12 h at 4 °C. After six washes with TBST, the membranes were incubated with HRP-conjugated Goat Anti-Mouse IgG (1,10,000 dilution) at room temperature for 1 h. Finally, proteins on membranes were detected by ECL chemiluminescence. CHO-K1-VH and CHO-K1-VL cell clones with detectable secretion were selected from each group for sequential expansion culture and cryopreservation.

### 2.7 Expression vector construction of PRV-scFv, protein expression and monoclonal cell screening

The *PRV-VH* and *PRV-VL* genes, selected based on their secretion and expression performance in CHO-K1 cells, were fused via a (Gly4Ser)3 linker. These genes were subsequently cloned into a pcDNA3.1(+)-C-6His vector, with codon optimization tailored for CHO-K1 cells, to create a PRV-scFv eukaryotic expression vector. Electroporation using a Lonza 4D-Nucleofector was employed to transfect 10 μg of the recombinant plasmid into 2 × 10^6^ CHO-K1 cells. Following electroporation, the cell suspension was plated onto a 6-well plate, and SFM4CHO medium was added to reach a final volume of 2 mL. The cells were then cultured at 37 °C in a 5% CO_2_ incubator without agitation. After 48 h, an appropriate volume of supernatant was collected for western blot analysis. Subsequently, at 72 h post-transfection, G418 at a concentration of 400 μg/mL was introduced to the cells to apply selection pressure. After 2–3 passages, the culture was gradually upscaled to shake-flask cultivation, with cells subcultured every 3 days at a density of 1.0 × 10^6^ cells/mL in a 50 mL culture volume. The cultivation took place at 37 °C in a 5% CO_2_ shaking incubator set at 120 rpm. Following 20 generations of selection under G418 pressure, the stable expression of the recombinant protein was evaluated through western blot analysis. Simultaneously, cells from the pooled population underwent monoclonal screening by seeding at a density of one cell per well in 96-well plates, followed by the addition of G418 for initial selection (Li et al., 2024a). The plates were then placed in a 37 °C incubator with 5% CO_2_. After 10 days, single-cell colonies were identified and observed in specific wells. Images were captured and stored using an inverted fluorescence microscope. The supernatant from selected wells was examined via western blot, and clones secreting PRV-scFv were individually named. A clone exhibiting high expression levels was selected for subsequent scale-up culture and cryopreservation for further experimentation.

### 2.8 The effect of fed-batch culture on the growth of engineered cell lines and recombinant protein expression

Our group implemented the established glucose feeding strategy for CHO-K1 cells by combining Cell Boost 5 (CB5) feed medium, SFM4CHO basal medium, and glucose, as detailed in Table 2 (Li et al., 2024a). CHO-K1-PRV-scFv-2^E8^ cell in logarithmic growth were subcultured at a density of 1.0 × 10^6^ cells/mL and divided into three experimental groups and one control group, each with a 100 mL culture volume. The cultures were maintained at 37 °C with 5% CO_2_ and an agitation speed of 120 rpm in a shaker incubator. The experimental groups followed the feeding regimen outlined in Table 2, while the control group remained unsupplemented. Starting from day 2, daily assessments of viable cell density and viability were conducted using a Countstar automated cell counter. Glucose levels were monitored with a glucose assay kit, and when concentrations dropped below 3.5 g/L, they were replenished to 7.0 g/L. Culture supernatants were harvested upon viability dropping below 60%, followed by centrifugation at 850 × g for 10 min to collect the supernatant. The recombinant protein content in the supernatant was analyzed through western blot.

**Table 2:** Feeding schemes.

Fed-batch time/(d)	0	5	7	9	11	13	15
CB5/(%)	2.5	5	7.5	7.5	5	5	5

### 2.9 Expression and purification of PRV-scFv protein

The cell culture supernatant underwent filtration through a 0.22 μm membrane filter to eliminate cellular debris, followed by ultrafiltration and concentration utilizing a Vivaflow® 50R, 10,000 MWCO Hydrosart ultrafiltration cassette. Subsequently, the concentrated sample was applied to a Ni Sepharose FF (NTA) column (Sunresin, Xi’an, China) that had been pre-equilibrated with a buffer comprising 20 mM imidazole, 20 mM phosphate, and 0.5 M NaCl at pH 7.4 using an ÄKTA purification system. Elution was carried out with buffers containing escalating imidazole concentrations. The eluted fractions underwent analysis via SDS-PAGE and western blotting to determine the recombinant protein content. Additionally, the protein concentration following ultrafiltration was assessed using the BCA Protein Assay Kit (Beyotime, Shanghai, China).

### 2.10 Antibody specific detection of PRV-scFv protein

The PRV-scFv protein was conjugated with horseradish peroxidase (HRP) following the manufacturer’s instructions of the HRP Rapid Labeling Kit (ECALBIO®, Wuhan, China). The ELISA plate supplied from the ELISA Blocking Kit, for Detecting Pseudorabies Virus gB Antibody, was equilibrated at room temperature for 30 min before use. The PRV-scFv-HRP conjugate was then diluted 25-fold and subjected to subsequent two-fold serial dilutions. Each well of the ELISA plate was incubated with 100 μL of the diluted conjugate at 37 °C for 1 h. The plate was then washed five times with TBST, and residual liquid was removed by blotting after the final wash. Positive and negative control groups were treated with 100 μL of positive serum and negative serum, respectively, and incubated at 37 °C for 30 min. Each well was then washed five times with PBST, and residual liquid was removed. Next, each well received 100 μL of enzyme-labeled substrate, followed incubation at 37 °C for 30 min. The wells were then washed five times with TBST and then blotting to dry. 50 μL of solution A was added to each well, immediately followed by 50 μL of solution B. The plate was then incubated at room temperature in the dark for 10 min to facilitate color development. The reaction was quenched by adding 50 μL of 2 mol/L H_2_SO_4_ to each well. Subsequently, the optical density at 630 nm (OD630 nm) was determined using microplate reader.

### 2.11 Cytotoxicity assessment

The cytotoxicity of different concentrations of PRV-scFv-2^E8^ protein was verified using the MTT assay. BHK-21 cells were exposed to different concentrations of PRV-scFv-2^E8^ protein for 24 h before an MTT assay was performed. The colorimetric change was measured at OD490 nm on a plate reader.

### 2.12 Assessing anti-PRV neutralizing effect of PRV-scFv-2E8 protein

BHK-21 cells in the logarithmic growth phase were plated at a density of 1 × 10^6^ cells per well in 6-well plates and cultured at 37 °C with 5% CO_2_ until they reached approximately 90% confluence. Subsequently, 100 μg and 300 μg of PRV-scFv-2^E8^ protein were mixed with an equal volume of 100 TCID_50_ of PRV and incubated at 37 °C for 30 min before exposure to the BHK-21 cells. The positive control group was inoculated with 100 TCID_50_ of PRV, whereas the blank control group received only culture medium without any additional treatment. After a 2-h incubation at 37 °C to facilitate viral infection, the supernatant was aspirated, and the cells were washed with serum-free DMEM to remove unbound virus. Subsequently, the cells were maintained in a 37 °C incubator with 5% CO_2_ for 24 h. TCID_50_ assays were employed to quantify infectious virus titers. Infected cells were subjected to three freeze–thaw cycles, followed by centrifugation to clarify the sample prior to supernatant collection. The supernatants were serially diluted 10-fold and subsequenctly inoculated on to BHK-21 cell monolayers cultured in 96-well plates. Cytopathic effects (CPE) were observed, and PRV titers were determined using the Reed and Muench method. Western blotting was employed to detect the expression of viral proteins. Harvested infected cells were lysed with RIPA lysis buffer (Solarbio, Beijing, China) to prepare whole-cell protein extract. The extracted proteins were analyzed using 10% SDS-PAGE and subsequently transferred onto PVDF membranes. The PVDF membranes were incubated with Pseudorabies Virus Polyclonal Antibody (1,800 dilution) and Beta Actin Monoclonal Antibody (1,5,000 dilution) for 12 h at 4 °C. After five washes with TBST, the membranes were incubated for 1 h at room temperature with HRP-conjugated Goat Anti-Mouse IgG and HRP-conjugated Goat Anti-Rabbit IgG (1,10,000 dilution), respectively. β-actin served as a loading control. Finally, proteins on the membranes were detected using ECL chemiluminescence (Bio-Rad, CA, United States).

### 2.13 Protective efficacy of PRV-scFv-2^E8^ protein in mice

Six to eight-week-old male BALB/c mice were acclimatized for 7 days and subsequently divided into six groups at random, five in each group. The prophylactic groups received retro-orbital injections of the PRV-scFv-2^E8^ protein. Two hours after administration, these groups were challenged with an intramuscular injection with 100 TCID_50_/500 μL of PRV. The therapeutic groups were challenged by intramuscular injection with 100 TCID_50_/500 μL of PRV. Two hours post-challenge, the PRV-scFv-2^E8^ protein were administered via retro-orbital venous injections. Additionally, a negative control group was infected through intramuscular injection with PBS, while a positive control group was infected with 100 TCID_50_/500 μL of PRV. Daily retro-orbital injections of PBS or PRV-scFv-2^E8^ were performed for 6 consecutive days, with the mice maintained under 2.5% isoflurane anesthesia during the procedures. The clinical health status of the mice was monitored daily. On day 7 of the post-infection, the mice were euthanized by gradual displacement with CO_2_ at a rate of 50% of the chamber volume per minute until respiratory arrest. Brain tissue and serum were collected for the assessment of PRV viral load. DNA was extracted from the tissue samples for the determination of PRV DNA load by quantitative polymerase chain reaction (qPCR), and viral titers were determined by the TCID_50_ assay. For qPCR, initial denaturation was performed at 95 °C for 30 s, followed by 40 cycles of denaturation at 95 °C for 5 s and annealing/extension at 55.6 °C for 34 s. The 20 μL reaction system consisted of 3 μL DNA template, 10 μL Premix Hs Taq (2×), 1 μL Probe, 0.5 μL forward primer, 0.5 μL reverse primer, 0.25 μL Rox Dye, and 4.75 μL DEPC-treated H_2_O. An PRV positive plasmid standard was used as a template under identical reaction conditions to generate a standard curve and equation. The probe sequence for PRV (5′ to 3′) was CCACGGCCGTCACGA, the forward primer (PRV-qF) was GTCAGGAGGCAGTTGTAGGC, and the reverse primer (PRV-qR) was CGGACCTCGTGAACTACCC. All qPCR reagents were obtained from Accurate Biology. For the TCID_50_ assay, brain tissues were homogenized in PBS. A survival curve was plotted based on the survival status of the mice.

### 2.14 Statistical analysis

All statistical analyses were performed on GraphPad Prism 8. The data was analyzed by either Student’s t-test or one-way ANOVA. The results are presented as the mean ± standard deviation (mean ± SD). Statistical significance was defined by the following *p*-values: *p < 0.05, **p < 0.01, and ***p < 0.001.

## 3 Results

### 3.1 Isolation and identification of *PRV-VH* and *PRV-VL* genes

**Figure 1:**
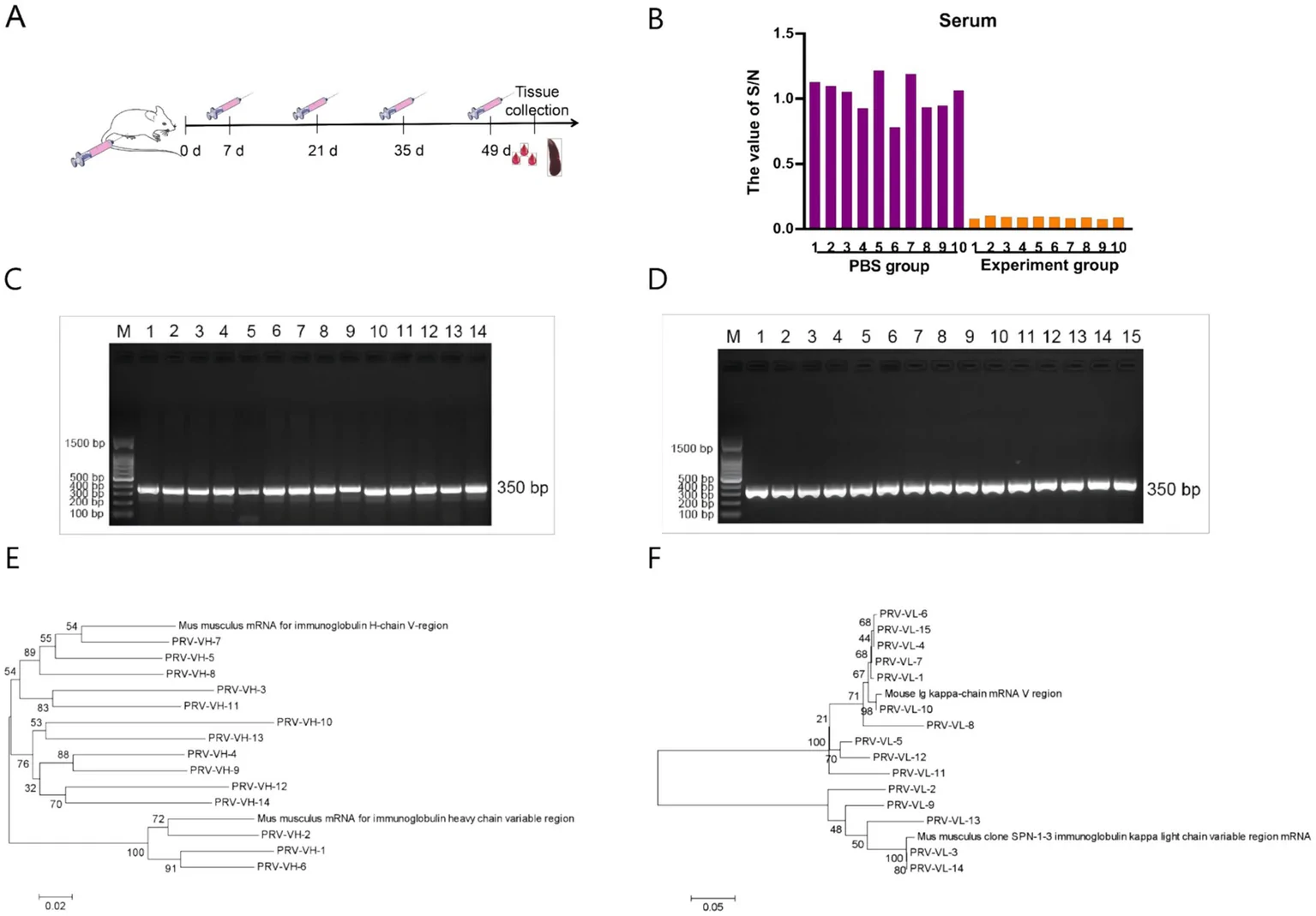
Isolation and identification of *PRV-VH* and *PRV-VL* genes. **(A)** A schematic summary of immunization method *in vivo*. **(B)** ELISA analysis of PRV gB antibody in immunized mouse serum, 10 mice per group. **(C,D)** Agarose gel electrophoresis of PCR amplified *PRV-VH* **(C)** and *PRV-VL* **(D)** gene products. The total RNA that isolated from the spleen of an immunized mouse was used as a template to RT-PCR with universal primers targeting the flanking the *PRV-VH* genes **(C**, lane 1–14, 350 bp**)**, *PRV-VL* genes **(D**, lane 1–15, 350 bp**)**. **(E,F)** The phylogenetic tree analysis of *PRV-VH* **(E)** and *PRV-VL* **(F)** gene sequence.

As shown in Figure 1A, after four immunizations, mice serum was obtained, and PRV-specific antibodies were identified using ELISA. Detectable antibodies were observed in all test groups PRV-specific IgB antibodies (Figure 1B). The *PRV-VH* and *PRV-VL* genes were amplified through PCR amplification, where the template cDNA was transcribed from the total RNA that isolated from the spleens of mice exhibiting positive PRV-specific gB antibodies. The amplified *PRV-VH* and *PRV-VL* genes were approximately 350 bp in length, aligning with the expected values (Figures 1C,D). Subsequently, the target genes were cloned into the pMD18-T vector, and after screening, positive clones were chosen for sequencing, yielding the gene sequences of *PRV-VH* and *PRV-VL*. BLAST analysis against the NCBI database confirmed that the obtained sequences share 99% homology with known *PRV-VH* and *PRV-VL* sequences. We also constructed neighbor-joining phylogenetic tree with MEGA 5.10 software (Figures 1E,F). Phylogenetic analysis revealed that the 14 acquired *PRV-VH* nucleotide sequences corresponded to the *VH* nucleotide sequences available on the NCBI database (GenBank: OZ390817.1 and AB160685.1). Similarly, the 15 obtained *PRV-VL* nucleotide sequences were analogous to the *VL* nucleotide sequences present on the NCBI database (GenBank: JX420690.1 and M36238.1). These results indicated that the *PRV-VH* and *PRV-VL* nucleotide sequences were successfully acquired in this investigation.

### 3.2 Construction and expression of eukaryotic expression vectors with *PRV-VH*/*PRV-VL* genes

**Figure 2:**
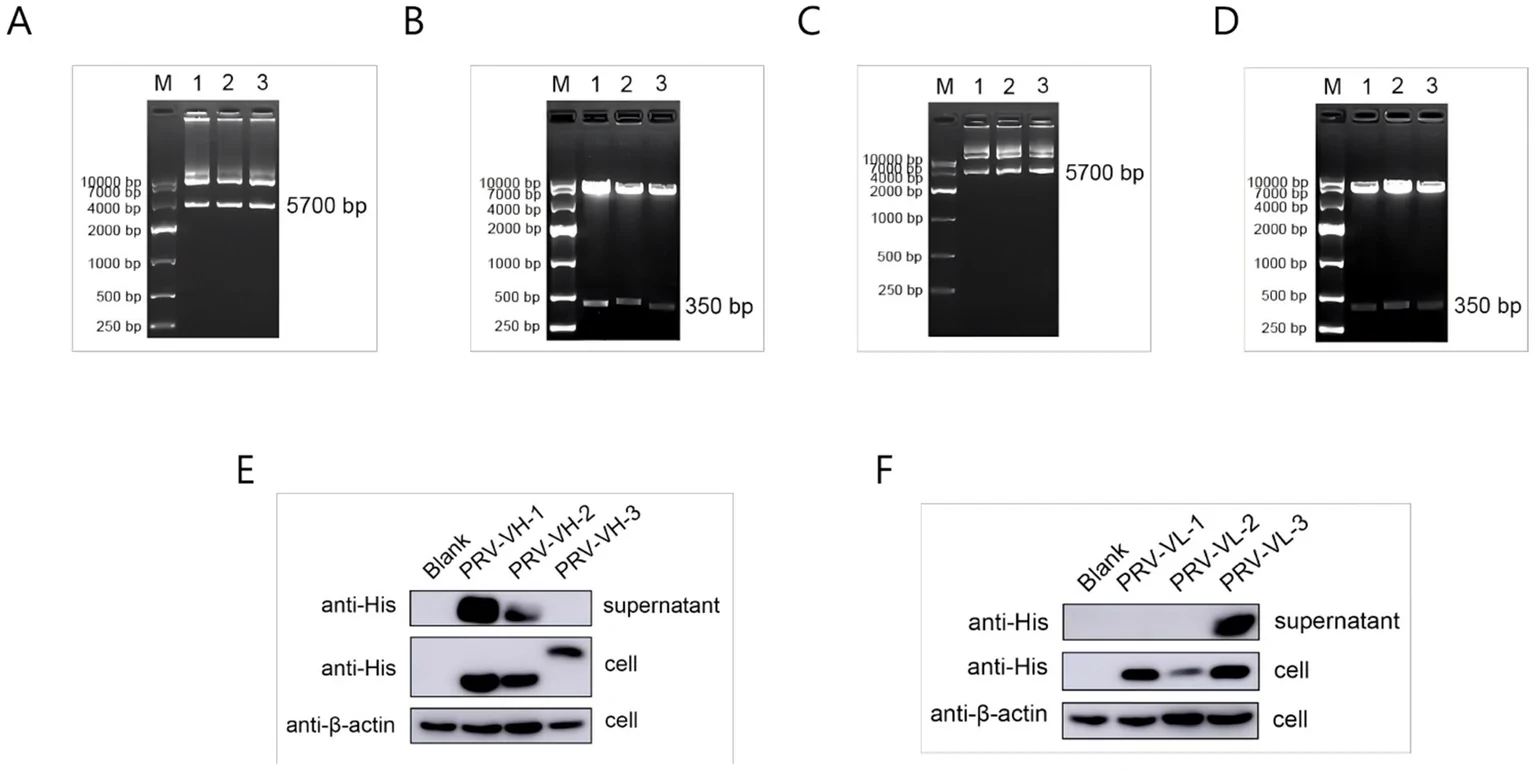
Generation of a stable cell line that produces PRV-VH and PRV-VL proteins. **(A**, **B)** Detection of PRV-VH recombinant plasmid by agarose gel electrophoresis **(A)** and restriction enzyme digestion **(B)**. The insert in these plasmids encodes the PRV-VH fused to a signal sequence and appended with a C-terminal 6 × His tag, all within the pcDNA3.1(+)-C-6His vector. Lane 1 represents the PRV-VH-1 recombinant plasmid, lane 2 represents the PRV-VH-2 recombinant plasmid, lane 3 represents the PRV-VH-3 recombinant plasmid. **(C,D)** Detection of PRV-VL recombinant plasmid through agarose gel electrophoresis **(C)** and restriction enzyme digestion **(D)**. The insert in these plasmids codes for the PRV-VL fused to a signal sequence and appended with a C-terminal 6 × His tag, all within the pcDNA3.1(+)-C-6His vector. Lane 1 represents the PRV-VL-1 recombinant plasmid, lane 2 represents the PRV-VL-2 recombinant plasmid, lane 3 represents the PRV-VL-3 recombinant plasmid. **(E,F)** PRV-VH protein and PRV-VL protein expression and western blot analysis. 10 μg of the recombinant plasmid and pcDNA3.1(+)-C-6His vector was transfected into 2 × 10^6^ CHO-K1 cells before extracting protein at 48 h post-transfection. Total protein levels in CHO-K1 cells were isolated by cold lysis buffer, and cell culture supernatants were quantified by western blotting. CHO-K1 cells transfected with the pcDNA3.1(+)-C-6His vector was served as the negative control. The relative levels of PRV-VH protein **(E)** and PRV-VL protein **(F)** were normalized to β-actin levels. Recombinant protein both present in the supernatant and cells, were detected using a His antibody via western blot analysis.

Three *PRV-VH* genes and three *PRV-VL* genes, selected based on phylogenetic analysis of their relatively evolutionary distances, were individually cloned into the pcDNA3.1(+)-C-6His vector. The resulting pcDNA3.1(+)-C-6His-VH and pcDNA3.1(+)-C-6His-VL plasmids were subjected to enzyme digestion with *BamH* I and *EcoR* I. Agarose gel electrophoresis confirmed that the digestion patterns for all constructs were as expected (Figures 2A–D). Subsequently, the expression of the recombinant plasmids pcDNA3.1(+)-C-6His-VH and pcDNA3.1(+)-C-6His-VL was verified in CHO-K1 cells. A total of 10 μg of plasmid was transfected into 2 × 10^6^ CHO-K1 cells. Following 48 h of expression, the cells were subjected to pressure selection and then cultured in shaking flasks for subsequent experiments. Western blot analysis was conducted at 48 h post-transfection, and the results demonstrated that the PRV-VH-1, PRV-VH-2, and PRV-VL-3 proteins were detected in both the supernatant and the cell lysates (Figure 2E). In contrast, the PRV-VH-3, PRV-VL-1, and PRV-VL-2 proteins were detected only in cell lysates (Figure 2F).

### 3.3 Design and generation of PRV-scFv proteins

**Figure 3:**
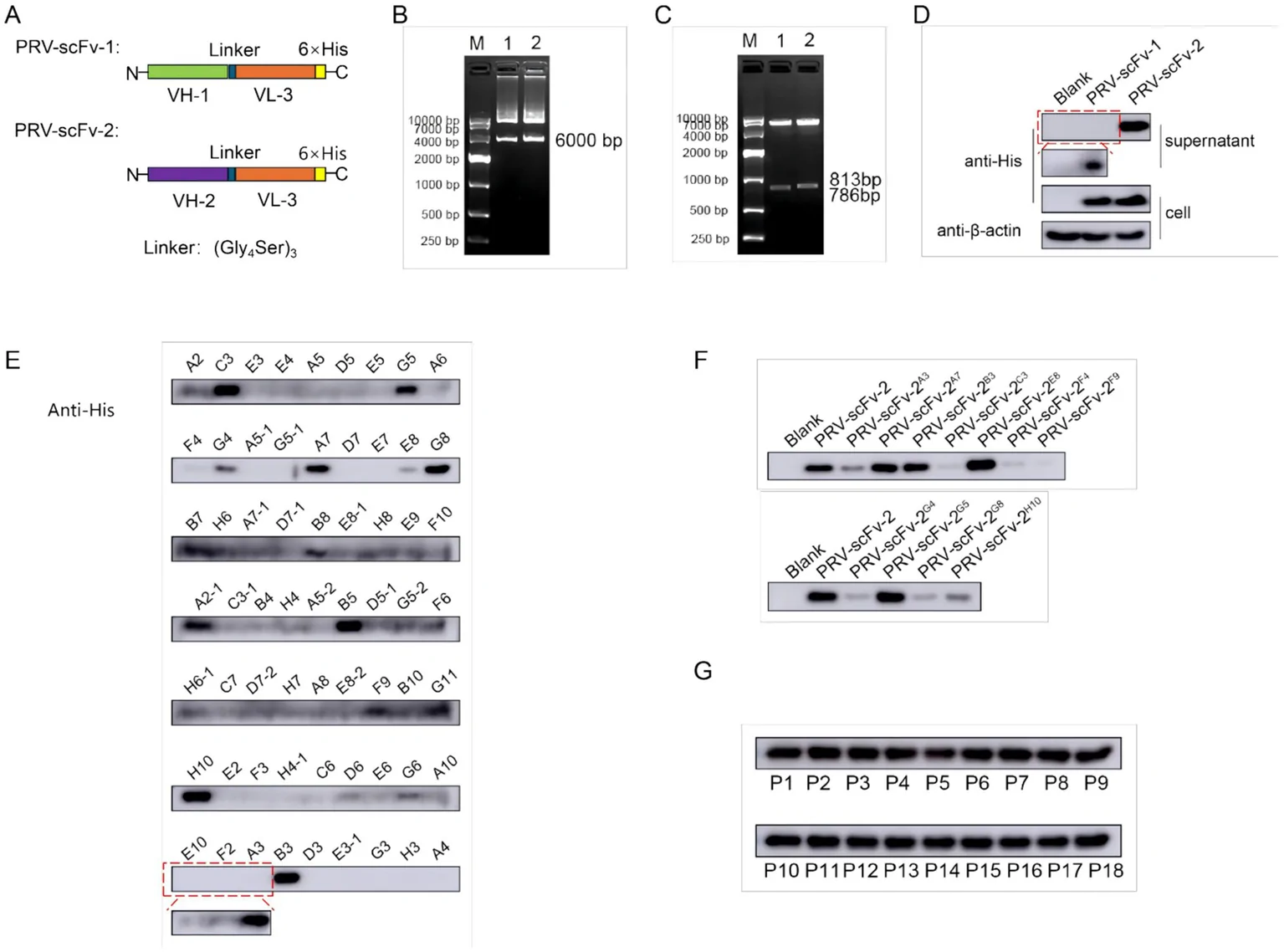
Construction, expression of PRV-scFv, screening of high-expression monoclonal cells and establishment of CHO-K1-PRV-scFv clone cells line **(A)** To engineer PRV-scFv, nucleotide coding sequences for VL and VH variable domains were connected by a (Gly4Ser)3 linker with a C-terminal 6 × His-tag in the pcDNA3.1(+)-C-6His vector. **(B,C)** Detection of PRV-scFv recombinant plasmid by agarose gel electrophoresis **(B)** and restriction enzyme digestion **(C)**. The insert in these plasmids encodes the PRV-scFv fused to a signal sequence and appended with a C-terminal 6 × His tag, all within the pcDNA3.1(+) vector. Lane 1 represents the PRV-scFv-1 recombinant plasmid, lane 2 represents the PRV-scFv-2 recombinant plasmid. **(D)** PRV-scFv protein expression and western blot analysis. 10 μg of the recombinant plasmid and pcDNA3.1(+)-C-6His vector was transfected into 2 × 10^6^ CHO-K1 cells before extracting protein at 48 h post-transfection. Total protein levels in CHO-K1 cells was isolated by cold lysis buffer, and cell culture supernatants were quantified by western blotting. CHO-K1 cells transfected with the pcDNA3.1(+)-C-6His vector was served as the negative control. The relative levels of PRV-scFv protein were normalized to β-actin levels. Recombinant protein, both present in the culture supernatant and cells, were detected using a His antibody via western blot analysis. **(E)** The monoclonal cells relative yield expressing PRV-scFv was detected by western blot. **(F)** The culture supernatant of monoclonal cells after expanded culture was detected by western blot. **(G)** Western blot examination of protein stability. PRV-scFv-2^E8^ protein at the indicated passage number post-stable drug selection for immunoblotting against His.

**Figure 4:**
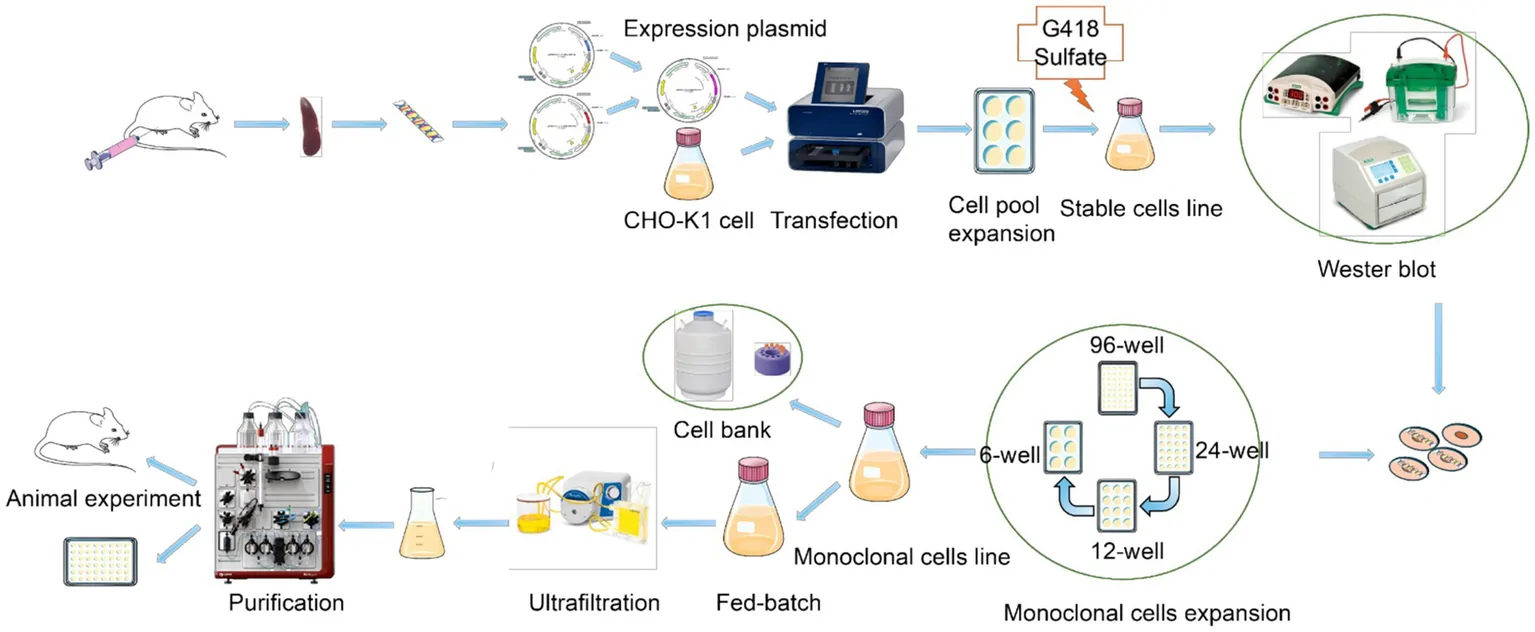
A stable cell line establishment flowchart.

To produce secreted PRV-scFv proteins, constructs were designed by fusing the N-terminal of *PRV-VH* variable domain genes with the C-terminal of *PRV-VL* variable domain genes using flexible glycine-rich (Gly4Ser)3 linkers (Figure 3A). Additionally, a 6 × His-tag was introduced to the C-terminal of fusion proteins using the pcDNA3.1(+)-C-6His vector to facilitate subsequent affinity purification. Analysis of the PRV-scFv recombinant plasmids through agarose gel electrophoresis revealed a distinct DNA band at approximately 6,000 bp, while restriction enzyme digestion confirmed bands at around 786 bp and 813 bp, consistent with the anticipated fragment sizes (Figures 3B,C). Furthermore, successful secretion of two plasmids was achieved in CHO-K1 suspension cells, with the fusion protein exhibiting a molecular weight of approximately 35 kDa, aligning with the predicted size of the protein (Figure 3D). Twenty-one monoclonal cell lines exhibiting high expression levels were isolated using the limiting dilution method. Among these, the PRV-scFv-2^E8^ monoclonal cells demonstrated the highest expression level and were selected for further experiments (Figures 3E,F). To determine whether the PRV-scFv-2^E8^ monoclonal cells could stably secrete the fusion protein in batch culture mode, stable cell lines expressing PRV-scFv-2^E8^ were established after 20 generations of continuous screening under selective pressure, as outlined in the flowchart in Figure 4. Western blot analysis was conducted on the culture supernatants from the 1st to the 20 th generations of the engineered cells. The results indicated that the fusion protein was consistently detectable across all generations of the CHO-K1 engineered cells, demonstrating that these cells maintained stable secretion efficiency throughout multiple generations (Figure 3G).

### 3.4 Fed-batch culture of the PRV-scFv-2^E8^ monoclonal cell lines

**Figure 5:**
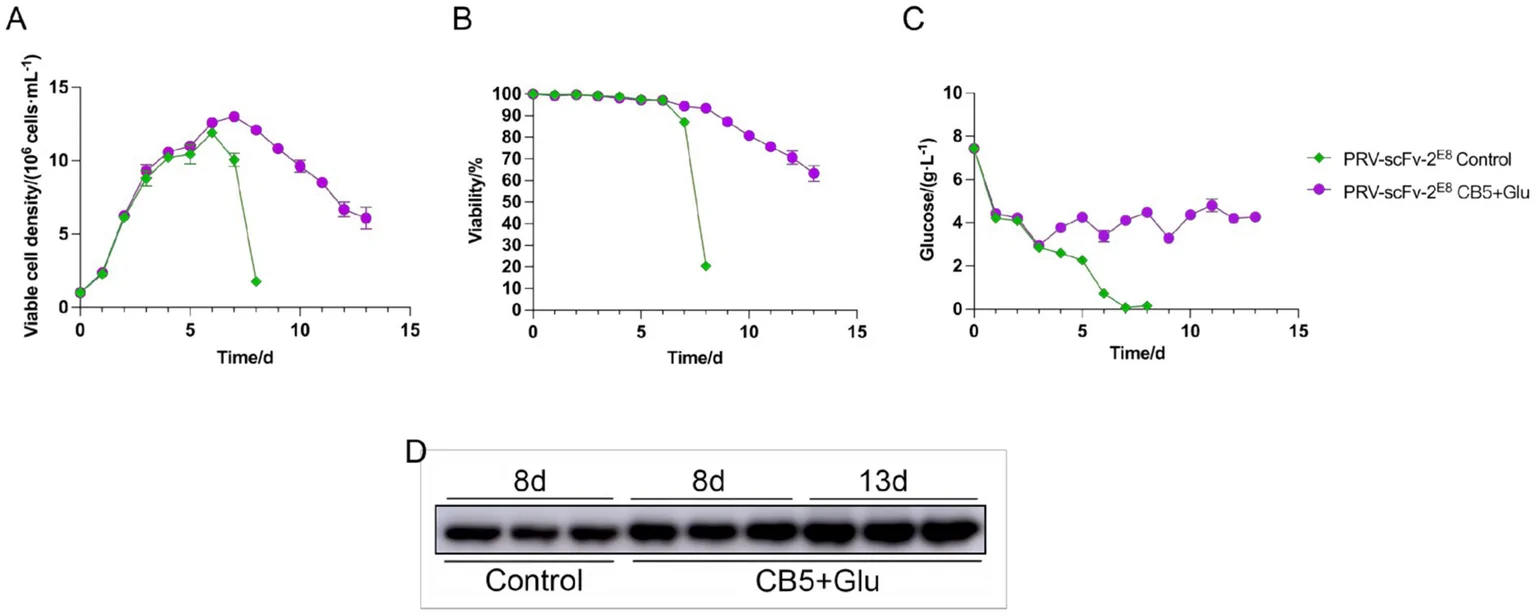
Influence of glucose and supplemented medium on CHO-K1-PRV-scFv-2^E8^ cell growth and protein expression. **(A)** Viable cell density over culture days. **(B)** Percentages of viable cells. **(C)** Glucose concentration in media. **(D)** Western blot analysis to reveal the protein expression of PRV-scFv-2^E8^ protein. When cell viability declines below 70%, it indicates that the environment is no longer suitable for cell growth. At this juncture, the supernatant is collected for the analysis of PRV-scFv-2^E8^ protein accumulation. On the 8th day, when the cell viability of the control group fell below 70%, the culture was terminated, and the supernatant was centrifuged for subsequent analysis. As a result, no supernatant from the control group was available for detection on the 13th day. Therefore, Western blot analysis was performed on the supernatant from the control group cells on the 8th day, as well as on the supernatant from the CB5 + Glu group cells on both the 8th and 13th days, to evaluate the effects of CB5 and glucose on the accumulation of PRV-scFv-2^E8^ protein.

To enhance the expression of PRV-scFv-2^E8^ protein in the engineered cell lines, all PRV-scFv-2^E8^ cell lines were cultured according to the previously established fed-batch culture protocol (Li et al., 2024a). The results demonstrated that the incorporation of glucose and CB5 into the culture medium extended the culture duration of these four genetically engineered cell lines by 5 days compared to the control group (Figures 5A–C). Furthermore, the addition of CB5 and glucose significantly increased the production of PRV-scFv-2^E8^ protein in the cell supernatant (Figure 5D). These findings suggest that the inclusion of glucose and CB5 can prolong the culture time of genetically engineered CHO cells while enhancing the secretory expression of PRV-scFv-2^E8^ protein.

### 3.5 Purification and concentration determination of PRV-scFv-2^E8^ protein

**Figure 6:**
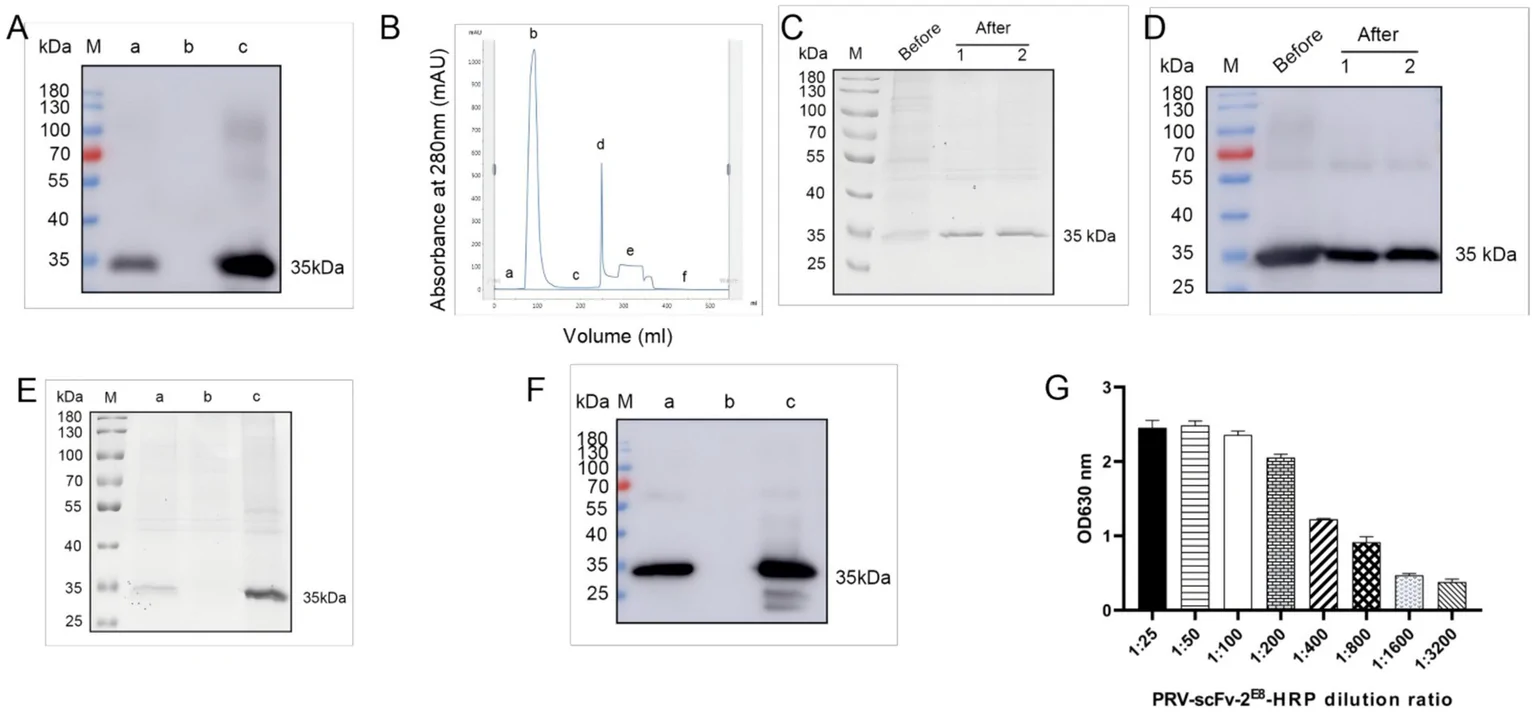
The purification identified by SDS-PAGE, western blot, and ELISA methods **(A)** Ultrafiltration results of the PRV-scFv-2^E8^ protein: (a) Before ultrafiltration sample, (b) ultrafiltration waste liquid, (c) after ultrafiltration sample. **(B)** Metal chelate chromatography profiles of the PRV-scFv-2^E8^ protein. The peaks represent the following fractions: (a) Wash fraction, (b) the fraction eluted with 250 mM imidazole, (c) repeated wash fraction, (d) the fraction eluted with 250 mM imidazole, (e) the fraction eluted with 500 mM imidazole, (f) final wash fraction. **(C,D)** The purification identified by SDS-PAGE **(C)** and western blotting **(D)**. Protein bands of the same molecular weight (35 kDa) appeared in pre-purification, post-purification 1 lanes, and 2 lanes. Western blot analysis using PRV-scFv-2^E8^ as the antigen, His-tagged mouse antibody as the primary antibody, and goat anti-mouse IgG-labeled HRP as the secondary antibody **(E,F)** Ultrafiltration results of the purified PRV-scFv-2^E8^ protein confirmed by SDS-PAGE **(E)** and western blotting **(F)**: (a) Sample before ultrafiltration of the purified PRV-scFv-2^E8^ protein, (b) ultrafiltration waste liquid, (c) sample after ultrafiltration of the purified PRV-scFv-2^E8^ protein. **(G)** Analysis of purified PRV-scFv-2^E8^ by ELISA. Detection of HRP labeled PRV-scFv-2^E8^ protein.

To improve the concentration and purification of the recombinant fusion protein, the cell culture supernatants were purified using a Vivaflow® TFF Cassette (Sartorius, Göttingen, Germany). Following ultrafiltration, the concentrations of the PRV-scFv-2^E8^ protein were significantly increased (Figure 6A). The His-tagged fusion proteins were subsequently purified with an ÄKTA pure chromatography system (Cytiva, Sweden) employing Ni Seplife FF (NTA) affinity chromatography columns (Sunresin, Xi’an, China). As illustrated in Figures 6B–D, the eluate obtained by eluting with 250 mM imidazole exhibited the highest yield of PRV-scFv-2^E8^ protein. After concentrating the collected eluate containing the PRV-scFv-2^E8^ protein (Figures 6E,F), the protein concentration was quantified at 2.6 mg/mL using a BCA assay. Subsequently, 2 mg of the PRV-scFv-2^E8^ protein was labeled with HRP for ELISA detection, and the results indicated that the PRV-scFv-2^E8^ protein could specifically bind to PRV (Figure 6G).

### 3.6 The PRV-scFv-2^E8^ protein effectively neutralizes PRV infection *in vitro*

**Figure 7:**
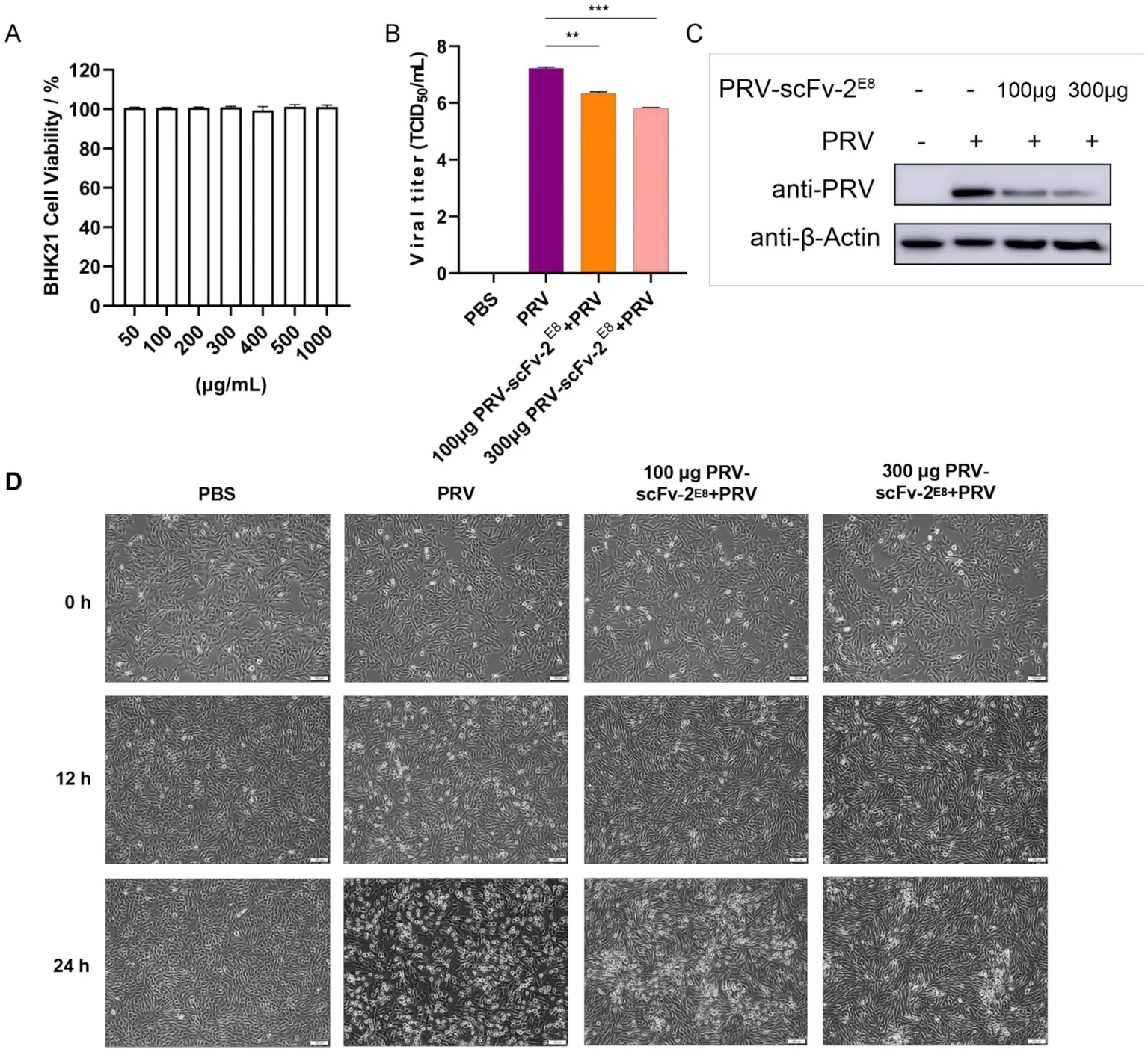
The PRV-scFv-2^E8^ protein neutralizes PRV infection *in vitro.* **(A)** BHK-21 cells were exposed to different concentrations of PRV-scFv-2^E8^ protein for 24 h before an MTT assay was performed. **(B,C)** 100 TCID_50_ of PRV was co-incubated with 100 μg and 300 μg PRV-scFv-2^E8^ protein at 37 °C for 30 min while exposed to a monolayer of BHK-21 cells. The cells were then washed with PBS and replenished with fresh culture media to each well. After 24 h, cells were subjected to PRV viral titer determined by TCID_50_ **(B)** and the PRV infection status was evaluated using specific antibodies against the PRV virus **(C)**. Data were presented as means ± SD, *n* = 3, statistical significance was defined as the following *p*-values: *p < 0.05, **p < 0.01, and ***p < 0.001. **(D)** Treated cells were also visualized for CPE under a bright field microscope at 10 × magnification. Scale bar, 100 μm.

To illustrate this in the context of PRV infection, we first assessed the cytotoxicity of the PRV-scFv-2^E8^ protein. As depicted in Figure 7A, the viability of BHK-21 cells remained above 80% after exposure to the PRV-scFv-2^E8^ protein, indicating no significant cytotoxicity. We incubated the PRV-scFv-2^E8^ protein with live PRV while exposing a monolayer of BHK-21 cells, followed by washing the cells. Subsequently, the cells were washed three times with DMEM medium, after which maintenance medium was applied. Viral neutralization activity is commonly assessed using functional assays such as TCID_50_ (Ko et al., 2023) and plaque reduction neutralization test (PRNT), which evaluate cell infection. The TCID_50_ method employed in this study represents a standard approach for measuring antibody efficacy against viral infection. The neutralizing activity of PRV-scFv-2^E8^ protein against PRV was determined using a TCID_50_ reduction assay. As shown in Figure 7B, the positive control group exhibited a titer of 7.225 log_10_ TCID_50_/mL. Treatment with 100 μg and 300 μg of PRV-scFv-2^E8^ protein reduced the titers to 6.338 and 5.817 log_10_ TCID_50_/mL. These results demonstrate a dose-dependent neutralizing effect of PRV-scFv-2^E8^ protein *in vitro*. The western blot analysis demonstrated that the marked reduction in the intensity of anti-PRV band after the treatment of 300 μg PRV-scFv-2^E8^ protein (Figure 7C). The bright field microscope images confirmed that treatment with PRV-scFv-2^E8^ protein reduced PRV-induced cytopathic effects (CPE), highlighting the antiviral effectiveness of the functionalized PRV-scFv-2^E8^ protein (Figure 7D).

### 3.7 PRV-scFv-2^E8^ protein inhibits the proliferation of PRV in tissue and rescues animals in a PRV lethal infection model

**Figure 8:**
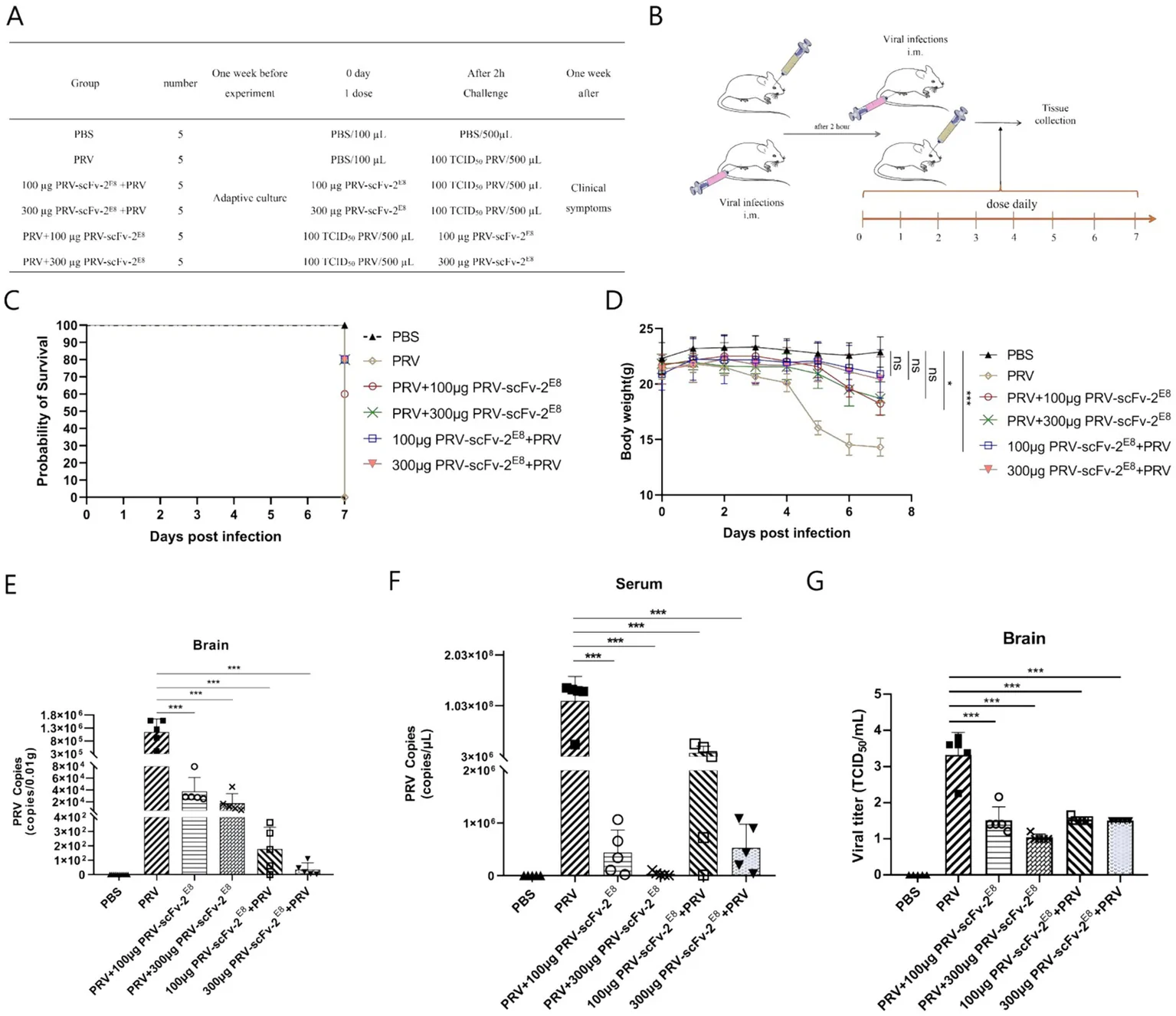
PRV-scFv-2^E8^ protein inhibits the proliferation of PRV in tissue and rescues animals in a PRV lethal infection model. **(A,B)** Experimental design for identifying the function of PRV-scFv in mice involved prophylactic and therapeutic groups. The prophylactic groups received PRV-scFv-2^E8^ protein via retro-orbital injection, followed by intramuscular PRV injection. The therapeutic groups were injected with PRV and then administered PRV-scFv-2^E8^ protein via retro-orbital injection. A negative control group received PBS intramuscularly, while a positive control group was injected with PRV **(B)**. **(C**–**G)** Mice were monitored daily for clinical survival **(C)** and weight loss **(D)**. On the 7 dpi or at experimental endpoint mice were euthanized, brain and serum collected for DNA extraction to assess **(E**,**F)** PRV viral load or tissues homogenized in PBS for 100 TCID_50_ in various organs **(G)**. Data were presented as means ± SD, *n* = 5, statistical significance was defined as the following *p*-values: *p < 0.05, **p < 0.01, and ***p < 0.001.

Although PRV is recognized as a neurotropic virus, it exhibits broad organ tropism, including the brain. To investigate the antiviral efficacy of the PRV-scFv-2^E8^ protein *in vivo*, we selected retro-orbital administration to ensure rapid systemic delivery and immediate distribution of the PRV-scFv-2^E8^ protein to the brain, thereby simulating intravenous infusion in an animal model. We applied both prophylactic and therapeutic administration route to a lethal PRV infection model (Figures 8A,B). The survival rate tracing indicated that administration of PRV-scFv-2^E8^ significantly enhanced clinical survival rate (Figure 8C). The untreated PRV-infected group exhibited significant body weight loss (Figure 8D). In contrast, no significant body weight loss was observed in the prophylactic group following 7 days of treatment with either 100 μg or 300 μg of PRV-scFv-2^E8^ (Figure 8D). Similarly, mice in the therapeutic group showed no significant changes in body weight after 7 days of treatment with either 100 μg or 300 μg of PRV-scFv-2^E8^ (Figure 8D). Then the brain and serum were collected for DNA extraction to assess PRV viral load (Figures 8E,F). The PRV viral loads in the treatment groups were dramatically lower than that of the untreated PRV-infected group, indicating the highly efficacious antiviral effect of PRV-scFv-2^E8^ protein *in vivo*. The 100 TCID_50_ results further demonstrated the significant antiviral effects in the brain and serum of PRV-infected mice in both administration ways of PRV-scFv-2^E8^ protein (Figure 8G).

## 4 Discussion

Pseudorabies virus infects various animals in the wild, with pigs serving as the primary reservoir. Mice and rabbits are the most susceptible experimental animals. Extensive research has been conducted on the neurotropism and latent infection of PRV (Bo et al., 2020). Since 2011, pseudorabies has resurfaced in multiple pig farms throughout China. The PRV strains responsible for this resurgence are distinguished by heightened virulence, broader transmission range, severe co-infections, and the failure of existing vaccines to offer complete protection, significantly hindering the national pseudorabies eradication initiative (Freuling et al., 2017; Nie et al., 2023; Sun et al., 2016; Wang et al., 2014; Wozniakowski and Samorek-Salamonowicz, 2015; Yin et al., 2025). Consequently, scFv characterized by their small molecular weight and robust tissue penetration capabilities that enable them to access lesion sites by crossing tissues or vascular barriers, present promising prospects for antiviral therapy (Wang et al., 2022; Yang et al., 2016). While scFv is predominantly produced in *Escherichia coli* (Yaghoobizadeh et al., 2023), the CHO cell expression system is also commonly utilized for scFv studies (Fogarasi et al., 2025). The CHO system allows high-level protein expression, facilitates appropriate post-translational modifications, and ensures proper folding/refolding, resulting in proteins that closely resemble their native counterparts and exhibit markedly enhanced biological activity compared to those generated in other expression platforms (Krebs et al., 2022; Ying et al., 2023). Although CHO-K1 cells facilitated correct assembly, its glycosylation profile—particularly the presence of *α*-1,3-galactose epitopes—poses a potential immunogenicity risk for *in vivo* therapeutic applications in swine or humans. This aspect differentiates our work from standard CHO-based expression reports; we assert that successful reformatting in CHO-K1 represents an intermediate rather than a final validation step. For PRV immunotherapy, subsequent glyco-engineering or substitution of the host cell line may be essential for producing fully functional, non-immunogenic antibodies.

CHO cell lines have emerged as the primary host system for the production of therapeutic recombinant proteins, owing to their capacity to generate complex proteins with post-translational modifications (PTMs), as well as their robustness, adaptability to suspension culture, and resistance to viral infections (Schulz et al., 2026). Consequently, CHO cells are extensively employed in manufacturing vaccines and pharmaceuticals (Sanchez-Martinez et al., 2024). In our study, the *PRV-VH* and *PRV-V*L genes were amplified from immunized mice spleens, showing significant homology with antibody variable region genes in the NCBI database through phylogenetic analysis, suggesting that the obtained gene sequences exhibit typical antibody variable region characteristics. Given the importance of expressing scFv antibodies to analyze their biological functions (Li et al., 2024b), recombinant protein expression was conducted using serum-free suspension-cultured CHO-K1 cells. Interestingly, some VH and VL proteins were secreted, while others were predominantly expressed intracellular. The discrepancy may stem from the sequence characteristics of the variable region genes, protein folding efficiency, and the recognition of secretion signal peptides. Based on this, the genes encoding the secreted VH and VL proteins were linked via a (Gly4Ser)3 linker to form scFv, and a eukaryotic expression vector was constructed. Subsequently, stable expression was achieved in CHO-K1 cells. After G418 screening and monoclonal selection, consistent stable expression levels were maintained through 20 consecutive passages. This outcome aligns with earlier findings by our research team (Li et al., 2025; Li et al., 2024a), confirming the successful development of a modified cell line proficient in secreting PRV-scFv-2^E8^ protein efficiently, thus establishing a basis for future large-scale production and application.

In optimizing cell culture processes, this study employed a fed-batch culture strategy. By supplementing the culture medium with CB5 and glucose, the culture duration of CHO-K1-PRV-scFv-2^E8^ cells was effectively extended, resulting in a significant increase in the expression level of the target protein. The findings suggest that a well-designed nutrient supplementation strategy not only sustains cell viability but also enhances the cumulative yield of recombinant proteins. This observation aligns with our research group’s prior studies, which demonstrated improvements in the expression of human serum albumin and EMCV multi-epitope candidate vaccines through the addition of glucose and CB5 in CHO cell culture media (Li et al., 2025; Li et al., 2024a). This provides a viable process reference for the industrial production of single-chain antibodies.

To achieve highly purified PRV-scFv-2^E8^ protein, the Vivaflow® TFF Cassette was utilized to concentrate and preliminarily purify the recombinant proteins. However, since this technology primarily relies on molecular size separation, the purity of the target protein may fall below 70%, leaving numerous impurities that could potentially impact the immunogenicity of the antibody. Therefore, further purification via affinity chromatography was conducted, ultimately producing a recombinant protein with high purity and a concentration of approximately 2.6 mg/mL. Following labeling the PRV-scFv-2^E8^ protein with HRP, ELISA confirmed its specific binding to PRV antigen, demonstrating favorable antigen recognition capability.

The study revealed a reduction in PRV virus titer with increased exposure to the PRV-scFv-2^E8^ protein. Western blot analysis indicated a significant decrease in anti-PRV band intensity after treatment with 300 μg of PRV-scFv-2^E8^ protein, highlighting its antiviral efficacy. This observation is consistent with previous research findings (Burton, 2023; Ku et al., 2015). However, it is important to recognize that infection inhibition assays alone do not fully clarify the specific molecular mechanisms involved in antibody-mediated neutralization. Further investigations, including epitope mapping, binding affinity analysis, and the examination of various stages of the viral replication cycle influenced by the antibody, are necessary for a comprehensive understanding of the underlying mechanisms.

Due to challenges associated with monoclonal antibodies (mAbs), including their large size, high production costs, low yield, mispairing, and production complexity, antibody fragments are viewed as the next generation of mAbs (Ahangarzadeh et al., 2020). Human viruses elicit strong, neutralizing antibody responses that serve as effective modalities for inhibiting viral infections (Lindner et al., 2019). Current research indicates that scFv can exert their virus-neutralizing effects before the virus enters host cells (Chang et al., 2021; Ren et al., 2024; Seyfizadeh et al., 2024). For instance, the construction of scFv targeting the spike protein of porcine epidemic diarrhea virus (a member of the coronavirus family) provides protection against viral infection in piglets (Zhang et al., 2019). Phoolcharoen et al. utilized a scFv with neutralizing potential against rabies, which was chemically conjugated with RVG (a 29-amino acid peptide) that specifically binds to nicotinic acetylcholine receptors in the central nervous system. This conjugate entered neuronal cells more efficiently than scFv alone, suggesting its potential as a novel therapeutic tool against rabies in the brain (Phoolcharoen et al., 2019). In our study, we found that the PRV-scFv-2^E8^ protein, at doses of 100 μg and 300 μg, effectively inhibited PRV-induced cytopathic effects (CPE) in BHK-21 cells, while also reducing viral titers and viral protein expression. These results demonstrate that PRV-scFv-2^E8^ protein can effectively block PRV infection in susceptible cells, aligning with findings from existing studies (Chang et al., 2021; Seyfizadeh et al., 2024). This provides a theoretical foundation for subsequent *in vivo* antiviral research.

The *in vivo* validation of our PRV-scFv-2^E8^ protein strategy further supports its potential. Experimental results showed that, irrespective of the administration schedule, the PRV-scFv-2^E8^ protein displayed significant antiviral effects in the brain tissue and serum of infected mice. This not only suggests the protein’s ability to effectively hinder PRV replication and spread *in vivo* but also indicates its potential to reach various infection sites through systemic circulation. Additionally, both administration schedules led to infected mice approaching body weights similar to those of the uninfected control group and significantly improved the clinical survival rate. These findings comprehensively illustrate the robust *in vivo* antiviral activity of the PRV-scFv-2^E8^ protein, underscoring its potential value for both preventing PRV infection and treating established infections. Nonetheless, this study has limitations. Firstly, while the scFv exhibited promising protective efficacy in mice, its protective potential in target animals like pigs necessitates further validation. Second, infection inhibition assays alone do not fully clarify the specific molecular mechanisms involved in antibody-mediated neutralization. Further investigations, including epitope mapping, binding affinity analysis, and the examination of various stages of the viral replication cycle influenced by the antibody. Third, although no antiviral biopharmaceuticals based on antibody fragments have been approved to date, considerable research efforts are focused on developing these therapeutics for viral infections. It is expected that, in the near future, these products will advance to clinical trials and may ultimately reach the market, establishing a significant class of therapeutic agents for infectious diseases, particularly those with viral origins.

In conclusion, we isolated the *PRV-VH* and *PRV-VL* genes from immunized mice spleens, connected them with a (Gly4Ser)3 linker to form PRV-scFv, and achieved stable expression in CHO-K1 cells grown in serum-free suspension culture. We identified a monoclonal cell line, designated CHO-K1-PRV-scFv-2^E8^ cell, that demonstrated high expression levels. This antibody effectively blocked PRV infection *in vitro*, reduced viral load *in vivo*, and enhanced the survival rate of infected mice, demonstrating its potential for antiviral use. These results introduce a promising antibody candidate for PRV antibody therapy and offer insights for the development of veterinary antibody treatments.

## Data Availability

The original contributions presented in the study are included in the article/supplementary material, further inquiries can be directed to the corresponding authors.
